# Reconstructing long-term dengue virus immunity in French Polynesia

**DOI:** 10.1371/journal.pntd.0010367

**Published:** 2022-10-03

**Authors:** Takahiro Nemoto, Maite Aubry, Yoann Teissier, Richard Paul, Van-Mai Cao-Lormeau, Henrik Salje, Simon Cauchemez

**Affiliations:** 1 Mathematical Modelling of Infectious Diseases Unit, Institut Pasteur, Université Paris Cité, UMR2000, CNRS, Paris, France; 2 Graduate School of Informatics, Kyoto University, Yoshida Hon-machi, Sakyo-ku, Kyoto, Japan; 3 Laboratory of research on Infectious Vector-borne diseases, Institut Louis Malardé, Papeete, French Polynesia; 4 Direction of Health, Ministry of Health, Papeete, French Polynesia; 5 Functional Genetics of Infectious Diseases Unit, Institut Pasteur, Université de Paris, UMR2000, CNRS, Paris, France; 6 Pasteur Kyoto International Joint Research Unit for Integrative Vaccinomics, Kyoto, Japan; 7 Department of Genetics, University of Cambridge, Cambridge, United Kingdom; Oregon Health and Science University, UNITED STATES

## Abstract

**Background:**

Understanding the underlying risk of infection by dengue virus from surveillance systems is complicated due to the complex nature of the disease. In particular, the probability of becoming severely sick is driven by serotype-specific infection histories as well as age; however, this has rarely been quantified. Island communities that have periodic outbreaks dominated by single serotypes provide an opportunity to disentangle the competing role of serotype, age and changes in surveillance systems in characterising disease risk.

**Methodology:**

We develop mathematical models to analyse 35 years of dengue surveillance (1979–2014) and seroprevalence studies from French Polynesia. We estimate the annual force of infection, serotype-specific reporting probabilities and changes in surveillance capabilities using the annual age and serotype-specific distribution of dengue.

**Principal findings:**

Eight dengue epidemics occurred between 1979 and 2014, with reporting probabilities for DENV-1 primary infections increasing from 3% to 5%. The reporting probability for DENV-1 secondary infections was 3.6 times that for primary infections. We also observed heterogeneity in reporting probabilities by serotype, with DENV-3 having the highest probability of being detected. Reporting probabilities declined with age after 14 y.o. Between 1979 and 2014, the proportion never infected declined from 70% to 23% while the proportion infected at least twice increased from 4.5% to 45%. By 2014, almost half of the population had acquired heterotypic immunity. The probability of an epidemic increased sharply with the estimated fraction of susceptibles among children.

**Conclusion/Significance:**

By analysing 35 years of dengue data in French Polynesia, we characterised key factors affecting the dissemination profile and reporting of dengue cases in an epidemiological context simplified by mono-serotypic circulation. Our analysis provides key estimates that can inform the study of dengue in more complex settings where the co-circulation of multiple serotypes can greatly complicate inference.

## Author summary

Characterising the true extent of dengue circulation and the level of population immunity is essential to assess the burden of disease, evaluate epidemic risk and organise prevention strategies against future epidemics. However, this is difficult in a context where most people who are infected by dengue virus (DENV) only have mild symptoms which may not be reported to surveillance systems. In this article, we develop a mathematical model to evaluate the fraction of unreported dengue infections from case data. The key idea is to introduce reporting probabilities that depend on the infecting serotype and the infection history of patients. These factors are known to contribute to variations in the severity of symptoms and hence the reporting probabilities, but have rarely been taken into account in model frameworks to study population immunity from the case data. Using the developed model, we study long-term dengue virus immunity in French Polynesia.

## Introduction

Dengue, which has four serotypes (DENV-1, DENV-2, DENV-3, DENV-4), is the most widespread mosquito-borne virus infection of humans. The vectors that transmit the dengue virus (DENV) are *Aedes* spp. mosquitoes, primarily *Aedes aegypti*, whose habitat is distributed in tropical and subtropical regions throughout the world. There are an estimated 3.97 billion people at risk of infection worldwide [1]. Most infections result in mild disease symptoms or are inapparent and are thus not reported to surveillance systems. However, certain patients suffer from severe symptoms, such as Dengue Hemorrhagic Fever (DHF) and Dengue Shock Syndrome (DSS) that have mortality rates of more than 20% if not properly treated [2]. Overall, 40,000 people died of dengue in 2017 [3]. Understanding risk factors for severe dengue is important to improve the clinical management of patients.

Many studies have shown evidence of higher incidence rates of severe dengue in secondary infected individuals [4,5]. Antibodies for the infecting serotype are produced upon infection. *In vitro*, these antibodies are observed to be cross reactive to the other serotypes: when antibody titres are not high enough, they enhance the entry of the other DENV serotypes into the host cells [6–8]. This phenomenon, called Antibody Dependent Enhancement (ADE), contributes to a severe disease outcome following infection. However, it is still not clear quantitatively and even qualitatively how severity is linked to the serotype, age, and the infection history of the patient [5]. Part of the challenge comes from the difficulty to reconstruct the infection history of patients in a context of high cross-reactivity between serotypes and in the common situation where multiple serotypes co-circulate.

**Fig 1:**
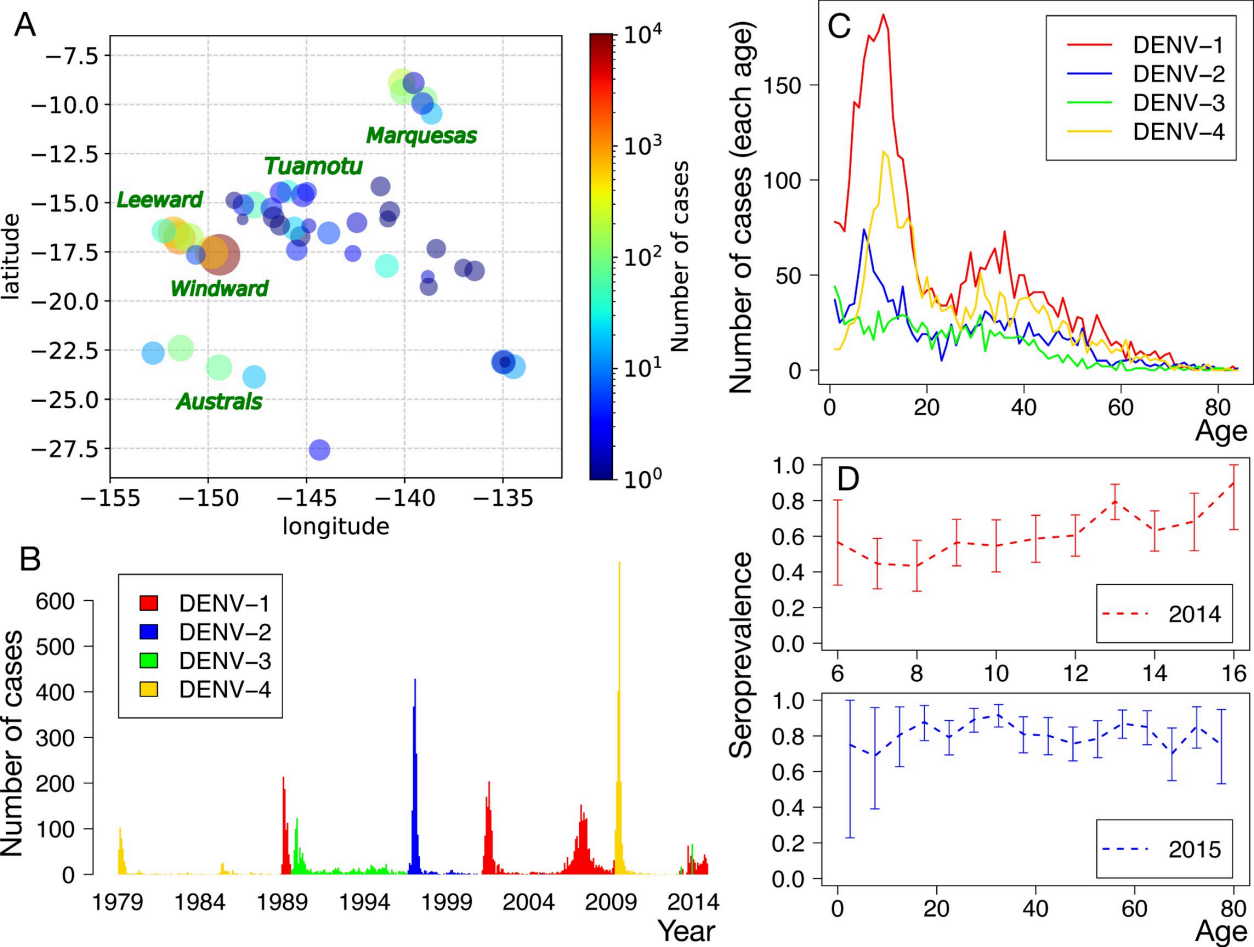
Epidemiology of dengue in French Polynesia (directly obtained from data). (**A)** Spatial distribution of the number of cases reported between 1979 and 2014, in the different islands of French Polynesia. Each circle represents the size of the population, where the radius is defined as 0.2log_10_P with the population size P. The colours of the circles represent the number of reported cases. (**B)** Monthly number of cases reported for DENV-1 to DENV-4. (**C)** Age distribution of cases, averaged over the period between 1979 and 2014. (**D)** The results of serological surveys (seroprevalence of antibodies against DENV) conducted in 2014 and 2015. Error bars indicate 95%-Confidence Intervals (CIs).

Here we aim to characterise how the history of dengue infection of a patient (i.e., when they were infected and by which serotype) may affect disease severity, considering over 30 years of surveillance as well as serological studies from French Polynesia, which consists of 119 islands, located in the middle of the Pacific Ocean [9] with 276 000 inhabitants (2017) (Fig 1A). We develop a mathematical model that describes how the circulation of dengue serotypes over multiple years has shaped the age-stratified immunity profile of the population, which in turn affects the reporting of infections in surveillance systems [10]. Under the assumption that reporting depends on disease severity, we use this framework to investigate the key drivers of disease severity and quantify their relative contributions. Compared to other settings such as in Southeast Asia, where multiple serotypes circulate each year, dengue epidemics in French Polynesia were mono-serotypic until only recently (2013–2014), making it possible to confidently reconstruct likely serotype-specific histories of infection, facilitating the evaluation of the impact of different infection histories on disease severity. We also investigate whether statistics that are estimated within our modelling framework, such as the fraction of susceptibles, can be used to anticipate the occurrence of an epidemic.

## Materials and methods

### Data

#### Surveillance data

The islands constituting French Polynesia are grouped into five administrative subdivisions: Windward, Leeward, Marquesas, Austral, and Tuamotu-Gambier (Fig 1). Since March 1975, Institut Louis Malardé in French Polynesia has received samples of suspected Dengue fever cases over the islands [11]. These samples have been analysed with different laboratory techniques, such as Haemagglutination Inhibition Assay (between 1975 and 1988), ELISA-IgM (1986–2003), isolation of DENV on mosquito C6/36 cell culture (1984–2005), and Reverse transcriptase Polymerase Chain Reaction (since 2000). Records include: the results of the tests (positive or negative), the serotype, the age of the patient, and the home community of the patient (from 1978).

In this work, we focus on the most inhabited subdivision, Windward, that includes three populated islands including Tahiti that alone accounts for 70% of the entire population of French Polynesia. We use the data from January 1979 to October 2014 and ignore data for children less than 1 year old to exclude the effect of maternal passive immunity [12]. We also use the demographic data from the census records of 1971, 1983, 1988, 1996, 2002, 2007 and 2012 [9]. The demographic data between these years are linearly interpolated from these censuses.

#### Seroprevalence data

Seroprevalence of antibodies against DENV was studied during May-June 2014 and during September-November 2015 in French Polynesia [13]. For the study conducted in 2014, 476 school children from primary and highschools in the most populous island (Tahiti) were recruited, while for the study in 2015, 700 people from the general population in the most inhabited subdivision (Windward) were recruited (Fig 1A). The blood samples of these participants were examined using a recombinant-antigen-based indirect ELISA against each serotype in the 2014 study [14,15] and microsphere immunoassay (MIA) with the same recombinant-antigens used for ELISA in 2015 study [16]. The antigens in this assay rely on the E3 domain of the flaviviruses, which minimizes cross-reactivity including with Zika virus.

### Mathematical model

Our model is composed of the following three parts: a model of dengue circulation in the population, a model of the surveillance system, and a model of serological surveys. We detail these models one by one and combine them in a likelihood function at the end.

#### Modelling dengue circulation and immunity in the population

The Force of Infection (FOI) characterises the transmission intensity of DENV and is defined as the per-capita rate at which susceptible individuals are infected. We denote *λ*_*i*_(*t*) the FOI for serotype *i* (*i* = 1,2,3,4) at time *t*. We assume that the FOI is a step function and define time periods during which the FOI is assumed to be constant, as shown in Table A in S1 Text (Note that these time periods correspond to epidemic periods except when a small number of dengue cases is reported. In these cases, the time periods simply correspond to one year.). The j-th time period is labelled *T*_*j*_ (*j* = 1,…,*J*), whose start time and duration are denoted *t*_*j*_ and *Δt*_*j*_, respectively. Since epidemics are mono-serotypic [11], there is only one serotype with a non-null FOI at any given time. Data are also available before 1979 but they are more limited [17]. They include the epidemic period and the circulating serotype. We thus set *λ*_*i*_(*t*) before 1979 to be non-zero only during the epidemic periods with the serotype *i*, where the magnitude of *λ*_*i*_(*t*) (if it is not zero) is constant across epidemics.

Using *λ*_*i*_(*t*), the immunity of the population is derived as follows. We denote by *x*(*t*, *a*|*λ*) the fraction of the population at age *a* (*a* = 1,2,…) that has never been infected by dengue before *t*. This population is susceptible to all dengue serotypes. As derived in Section A in S1 Text, *x*(*t*, *a*|*λ*) is computed as [18]

$$x(t,a|\lambda )=exp[-\int_{0}^{a}ds\sum_{i=1}^{4}\lambda_{i}(t-s)],$$ (1)

Similarly, we denote by *y*_*i*_(*t*, *a*) the fraction of population at age *a* who has been infected once by serotype *i* before *t* but is still susceptible to other serotypes [18]:

$$y_{i}(t,a|\lambda )=x(t,a)\{exp[\int_{0}^{a}ds\lambda_{i}(t-s)]-1\}.$$ (2)

See Section A in S1 Text for the derivation. We then denote by *z*(*t*, *a*) the fraction of population at age *a* who has been infected more than once before time *t*. We assume that this population is not susceptible to any dengue serotype based on [19] that showed tertiary and quaternary infections are negligible. Note that $x(t,a|\lambda )+\sum_{i=1}^{4}y_{i}(t,a|\lambda )+z(t,a|\lambda )=1$. The framework to compute these fractions of population based on FOI is known as the sero catalytic model and has been used in many studies [18, 20–28].

Denote $I_{p}^{i,j}(a|\lambda )$ and $I_{s}^{i,j}(a|\lambda )$ the number of individuals newly infected by serotype *i* (*i* = 1,2,3,4) at age *a* during time period *T*_*j*_ for primary and secondary infections, respectively. These are computed as

$$I_{p}^{i,j}(a|\lambda )=\int_{t_{j}}^{t_{j}+\Delta t_{j}}\lambda_{i}(t)x(t,a)P(t,a)dt,$$ (3)

$$I_{s}^{i,j}(a|\lambda )=\int_{t_{j}}^{t_{j}+\Delta t_{j}}\lambda_{i}(t)\sum_{i^{\prime }\neq i}y_{i^{\prime }}(t,a)P(t,a)\;dt$$ (4)

for *i* = 1,…,4, where *P*(*t*, *a*) is the number of individuals aged *a* at time *t*. Practically, to evaluate these integrals (and the integrals appearing below), we use the Euler method with step size 4. Assuming that there are no tertiary infections (and higher), the total number of newly infected individuals at age *a* during time period *T*_*j*_ is given as $I^{j}(a|\lambda )=I_{p}^{j}(a|\lambda )+I_{s}^{j}(a|\lambda )$, where $I_{p}^{j}(a|\lambda )=\sum_{i=1}^{4}I_{p}^{i,j}(a|\lambda )$ and $I_{s}^{j}(a|\lambda )=\sum_{i=1}^{4}I_{s}^{i,j}(a|\lambda )$.

Finally, we consider the effect of cross protection [29] for secondary infections. Upon primary infection, antibody levels rise and then slowly reduce over time [30]. It was estimated that antibodies cross-protect the patient from other DENV serotypes for an average of 2 years after the primary infection, followed by a period when antibodies may cause ADE [29]. (Note that reporting probabilities in our model are for any symptomatic cases: we implicitly assume that ADE can result in more symptomatic dengue cases, rather than just result in more severe dengue cases.) We assume that cross protection does not alter the immune profile of the population, *x*(*t*, *a*|*λ*) and *y*_*i*_(*t*, *a*|*λ*), but only affects the detected number of infected people (*i*.*e*., we assume that those who are protected become asymptomatic, when they are infected, due to cross protection). We denote *δx*_*i*_(*t*, *a*) the fraction of population aged *a* at time *t* who has been infected by DENV-*i* as primary infections in the past 2 years. Using this quantity, we replace *y*_*i*′_(*t*, *a*|*λ*) in $I_{s}^{i,j}(a|\lambda )$ by $y_{i^{\prime }}(t,a|\lambda )-\delta x_{i^{\prime }}(t,a)$, which is the true fraction of susceptible for the secondary infections by DENV-*j* (*j*≠*i*), excluding those who are protected due to cross protection. (Precisely, some of the individuals who are once in *δx*_*i*_(*t*, *a*) can be secondarily infected during the 2 years without symptoms. We ignore them by assuming that their contribution is small.) Once we take into account cross protections, the number of secondary infected individuals of age *a* during time period *T*_*j*_ by DENV-*i* is:

$$\bar{I_{s}^{i,j}}(a|\lambda )=\int_{t_{j}}^{t_{j}+\Delta t_{j}}\lambda_{i}(t)\sum_{i^{\prime }\neq i}\left[y_{i^{\prime }}(t,a|\lambda )-\delta x_{i^{\prime }}(t,a)]P(t,a\right)\;dt.$$ (5)

#### Modelling the surveillance system

Not all infected individuals are reported to the surveillance system. Some may be asymptomatic and not seek medical attention. Others may consult a medical doctor and yet not be recommended for a dengue test. Reporting of infected individuals can depend on

1. the age of the individual (infants, adolescents, or adults)
2. the time when the individual is infected
3. the infection history of the individual (primary infection or secondary infection)
4. the infecting serotype (DENV-1, DENV-2, DENV-3, DENV-4)

In order to take into account these different factors, we define the reporting probability *Φ*(*t*, *a*, *i*, *s*) as a function of *t* (the time of infections), *a* (the age of the infected individual), *i* (the infecting serotype (*i* = 1,2,3,4)), and *s* (an indicator of primary (*s* = 1) or secondary infections (*s* = 2)). To reduce the number of free parameters in the model, we assume the following dependency structure for reporting probabilities:

Φ(t,a,i,s)=T(t)A(a)φ(i,s), (6)

where *φ*(*i*, *s*) is the relative strength of the reporting probabilities, compared with primary infections of DENV-1 (i.e., *φ*(1,1) = 1). We assume that the age factor *A*(*a*) is constant over age groups 1–4 y.o., 5–9 y.o., 10–14 y.o., 15+, where we set the value for 5–9 y.o. at 1. Finally, the time factor *T*(*t*) is assumed to be constant during the following time periods 1979–1985, 1986–1990, 1991–1995, 1996–2000, 2001–2004, 2005–2008, 2009–2014, and increase between them. *T*(*t*) is the reporting probability for *i* = *j* = 1 and for the age group of 5–10 y.o..

The predicted number of reported cases of age *a* during time interval *T*_*j*_ is modelled using negative binomial distributions with mean

$$m^{j}(a|\lambda ,\Phi )=\sum_{i=1}^{4}I_{p}^{i,j}(a|\lambda )\Phi (t_{j},a,i,1)+\sum_{i=1}^{4}\bar{I_{s}^{i,j}}(a|\lambda )\Phi (t_{j},a,i,2)$$ (7)

and dispersion parameter

$$\alpha^{j}(a|\lambda ,\Phi ,k)=m^{j}(a{\left|\lambda ,\Phi \right)}^{k},$$ (8)

where *k* is a fitting parameter. (Note that, using the average and the dispersion parameter, the variance is expressed as *m*+*m*^2^/*α*. In the large *k* limit, the variance converges to *m* and the distribution becomes the Poisson distribution.)

Our model of the surveillance system is based on [10]. Technically, the differences from the previous work are that (i) the reporting probabilities in our model fully characterise how reporting probabilities vary with serotype, age, and the infection history of the patients, and (ii) the previous work used the Poisson distribution for the prediction, which led to narrow confidence intervals that may not reflect underlying statistical uncertainty, while we use the negative binomial distribution, from which broader confidence intervals can be produced.

#### Modelling serological surveys

For the *m*-th serological survey, we denote by *t*_*m*_, *n*_*m*_(*a*), and *C*_sero,*m*_(*a*) the time when the survey was performed, the number of participants of age *a*, and the number of participants of age *a* with antibodies (*t*_1_ = 2014.42 and *t*_2_ = 2015.75). From the reconstructed immune profile of the population, the seroprevalence of antibodies against any dengue serotypes *S*(*t*_*m*_, *a*|*λ*) is calculated as

$$S(t_{m},a|\lambda )=1-x(t_{m},a|\lambda ).$$ (9)

We assume that the probability that the participants have the antibodies follows a Binomial distribution with the number of trial *n*_*m*_(*a*) and a success probability *S*(*t*_*m*_, *a*|*λ*).

#### Likelihood function and Bayesian inference

We denote by $C_{\mathrm{rep}}^{j}(a)$ the number of reported cases for age *a* and for time interval *T*_*j*_. The likelihood function *L*(*C*_rep_, *C*_sero_|*λ*, *Φ*, *k*) is the product of the contributions from case reporting and from serological surveys:

$$L(C_{\mathrm{rep}},C_{\mathrm{sero}}|\lambda ,\Phi ,k)=\prod_{j}\prod_{a=1}^{80}\mathrm{negbin}[C_{\mathrm{rep}}^{j}(a)|m^{j}(a|\lambda ,\Phi ),\alpha^{j}(a|\lambda ,\Phi ,k)]$$

$$\times \prod_{m}\prod_{a=1}^{80}\mathrm{bin}[C_{\mathrm{sero},m}(a)|n_{m}(a),S(t_{m},a|\lambda )],$$ (10)

where negbin[*C*|*x*, *y*] is the negative binomial distribution function of *C* with the mean *x* and the dispersion parameter *y*, bin[*C*|*x*, *y*] is the binomial distribution function of *C* with the number of trial *x* and a success probability *y*. We use a Bayesian Markov Chain Monte Carlo (MCMC) framework and fit the model to the data. We determine the parameters *λ*, *Φ*, *k* using a uniform prior. We perform 450 000 iterations with 30 000 iterations as burn-in. We define a 95% credible interval using 2.5% and 97.5% percentiles of the posterior distribution. See Section B in S1 Text for more details of this inference and visualisations of the results.

#### Sensitivity analyses

We conducted analyses to understand the sensitivity of our model to the cross-immunity assumptions and to time varying reporting. In the Supporting Information (S1 Text), we show the results of our model (i) without cross immunity (Fig C in S1 Text), (ii) with a less varying reporting probability over the surveillance period (Figs E and F in S1 Text), and (iii) with a constant reporting probability over the surveillance period (Fig D in S1 Text).

#### Evaluation of the inferential framework using synthetic data

In order to evaluate the accuracy of our Bayesian inference to infer the model parameters, we first generate synthetic data by using the model described above. That is, for a fixed parameter set (the force of infection *λ*_*i*_(*t*), the reporting probability *Φ*(*t*, *a*, *i*, *s*), and the exponent *k* in the dispersion parameter), we simulate the number of reported cases $C_{\mathrm{rep}}^{j}(a)$ and the number of seropositive *C*_sero,*m*_(*a*) using the negative binomial and the binomial distributions (in Eq (10)). Next, from these simulated data $C_{\mathrm{rep}}^{j}(a)$ and *C*_sero,*m*_(*a*), we infer the parameter set *λ*_*i*_(*t*), *Φ*(*t*, *a*, *i*, *s*) and *k* using our inferential framework. By comparing the true model parameters with the inferred ones, we evaluate the accuracy of our Bayesian inference. We use the median of the inferred parameters from the real data as the model parameters to generate synthetic data.

## Results

### Epidemiology of dengue in French Polynesia

The spatial distribution of the reported cases is reported in Fig 1A. It shows that the reported cases are concentrated in Windward islands. Eight epidemics occurred during the 35 years between 1979 and 2014 (Fig 1B). Note that, in French Polynesia, mono-serotype epidemics had been recorded since 1944 until the recent heterotypic outbreak of 2013/2014. Until the emergence of Zika in 2013, the only arboviruses to be identified as circulating were the DENV serotypes [31]. Children represented most of the observed dengue cases (Fig 1C). The seroprevalence of antibodies against DENV is around 0.8 for the general population while it is around 0.6 for children, indicating children were more susceptible to DENV at the time of the serostudy (Fig 1D).

### Reporting probabilities

**Fig 2:**
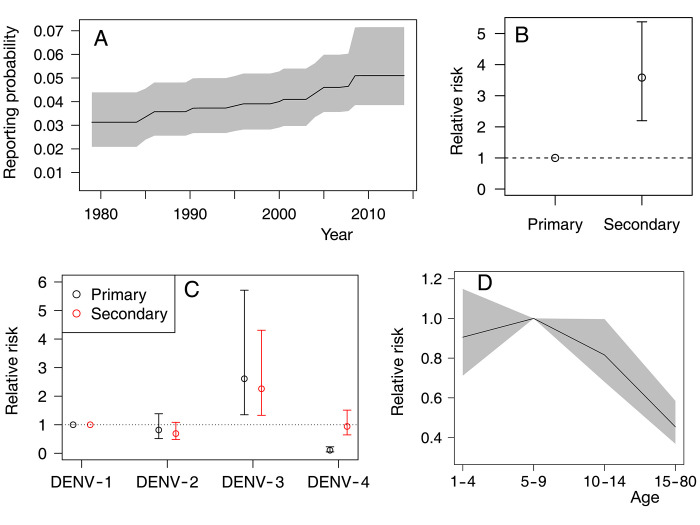
Estimated reporting probabilities (obtained from our model). **(A)** Reporting probability of primary infections by DENV-1 as a function of time. The grey shaded area shows 95%-CI. **(B)** Relative strength of the reporting probabilities of secondary infections (DENV-1) compared with primary infections (DENV-1). **(C)** Comparison of the reporting probabilities for different serotypes, for primary (black circle) and secondary (red circle) infections. The reference group is primary Serotype 1 infection for primary infections and secondary Serotype 1 infection for secondary infections. **(D)** Variations of the reporting probability with the age group, considering individuals aged 5–9 year old as the reference group. See Section B in S1 Text for the mathematical definitions of the relative risks plotted in these panels.

Under the assumption that the surveillance system improves over time, we estimate that, in French Polynesia, the probability of reporting a primary infection by DENV-1 increased from 3.13% in 1979 (95%-CI 2.09%-4.39%) to 5.10% in 2014 (95%-CI 3.86%-7.15%) (Fig 2A). The reporting probability for secondary infections by DENV-1 was 3.58 (95%-CI 2.20–5.38) times larger than that for primary infections by the same serotype (Fig 2B), reflecting a general result that secondary infections are more likely to be reported due to increased symptom severity [5]. Fig 2C shows how the reporting probability changes with the serotype and whether it is a primary and secondary infection, considering DENV-1 as the reference serotype. For primary infections, reporting probabilities for DENV-2, DENV-3 and DENV-4 are 0.82 (95%-CI 0.51–1.38), 2.61 (95%-CI 1.35–5.71) and 0.11 (95%-CI 0.05–0.23) times larger than that for DENV-1. For secondary infections, the reporting probabilities are 0.69 (95%-CI 0.48–1.08), 2.26 (95%-CI 1.33–4.31) and 0.94 (95%-CI 0.64–1.51) times larger for DENV-2, DENV-3 and DENV-4 respectively than the one for a secondary DENV-1 infection. Note that the reporting probabilities for DENV-4 are small for primary infections. This is in line with observations in Thailand [30, 32] and Nicaragua [33], where DENV-4 was shown to be circulating even though there were not many reported cases of that serotype. We also found that cases older than 14 years old are 0.45 (95%-CI 0.37–0.58) less likely to be reported than those aged 5–9 year old (Fig 2C). Reporting probabilities for children aged 1–4 y.o. and 10–14 y.o. are of the same order as those for children aged 5–9 y.o.

### FOI and immunity in the population

**Fig 3:**
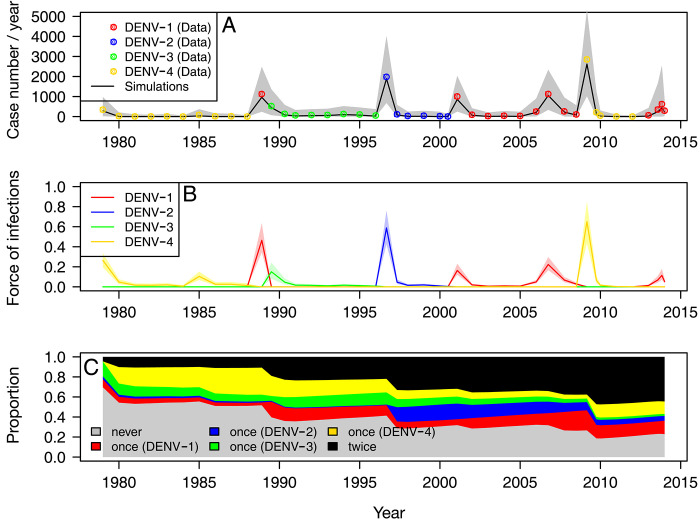
Estimated FOI and immunity (obtained from our model). **(A)** The observed (dots) and expected (line) number of cases reported annually. Shaded area represents 95%-CI. **(B)** Fitted FOI for the four serotypes. **(C)** Average immunity profile of the population. The grey area shows the fraction of the population that were never infected, averaged over age groups. Red, blue, green, and yellow areas represent the fraction of the population who have been infected once by a serotype i (before the time we consider), where i = 1,2,3,4 correspond to red, blue, green, yellow, respectively. Black area represents the fraction of the population who have been infected more than once (before the time we consider).

Next, we compare the observed and expected number of reported cases per year in Fig 3A, while estimated FOI are presented in Fig 3B. The FOI and the number of reported cases show qualitatively similar peaks, but some details are different. For example, the largest epidemics in terms of reported cases occurred in 2009 and was due to DENV-4 (2853 / year), followed by the second largest in 1996 due to DENV-2 (1983 / year). In terms of FOI, the extent of these two epidemics is more or less the same, with an FOI at 0.65 (95%-CI 0.44–0.85) for the 2009 DENV-4 epidemic and 0.59 (95%-CI 0.43–0.76) for the 1996 DENV-2 epidemic. This could be because (i) the quality of surveillance systems improved over time as seen from Fig 2A, and also because (ii) the secondary-infection reporting probabilities for DENV-2 (relative risk: 0.69) are smaller than those for DENV-4 (0.94) and DENV-1 (1). The same argument can also explain the relatively small FOI observed for DENV-1 in the epidemics occurring after 2000.

Using the estimated parameters, we reconstruct the immunity profile of the population during the surveillance period and plot it in Fig 3C. The graph shows that the immunity profile changed over time. During the course of the surveillance period (1979–2014), the proportion that were never infected declined from 0.70 to 0.23 while the proportion that were at least twice infected increased from 0.045 to 0.45 at the end of the period (2014). The general population has experienced several dengue epidemics during the 35 years of surveillance. As a result, almost half of the population has acquired heterotypic immunity.

### Age distribution and immunity

**Fig 4:**
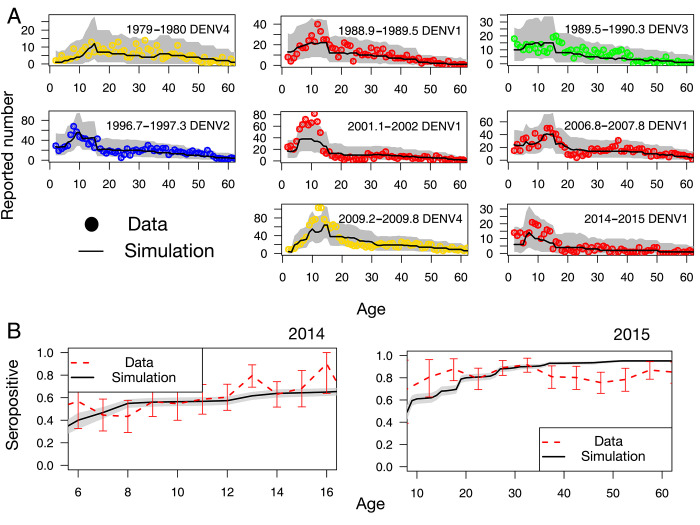
Observed and expected age distribution. **(A)** Age distributions of the reported case numbers during the epidemic periods (red, blue, green, yellow circles correspond to the serotype 1, 2, 3, 4, respectively). **(B)** The seroprevalence of antibodies against DENV obtained from the serological survey (red dashed lines with error bars). In both (A) and (B), black solid lines give model predictions, with 95%-CI represented by the grey shaded areas (95%-CI).

In Fig 4A, we show the age distribution of the number of reported cases for all epidemics during the surveillance period. Overall, the data points fall inside the 95%-CI, showing that our model captures well the age structure of the immunity of the population. The shapes of certain distributions in Fig 4A can be qualitatively understood based on the severity of secondary infections as follows. The age distributions of the epidemics during 1988–1989, 1996–1997, 2001–2002, and 2009 have a plateau (or a peak) and to the left side of this plateau, a relatively small number of reported cases is observed. The plateaus start around 10, 6, 5, and 8 years of age for the epidemics during 1988–1989, 1996–1997, 2001–2002 and 2009, respectively. The children whose ages are less than these plateau ages did not experience the previous epidemic. This means that they had less risk of having secondary infections, so that it is consistent with the observation that the age group to the left side of the plateau had less reported numbers. In Fig 4B, we compare the results of the serological survey (the seroprevalence of antibodies against DENV in 2014 and 2015) [13] with the model predictions using the fitted parameters. For the 2014 survey, most of the model predictions fall within 95%-CI, while for the 2015 survey, small systematic deviations in some age groups are observed.

### Relation between the proportion of susceptibles and FOI

**Fig 5:**
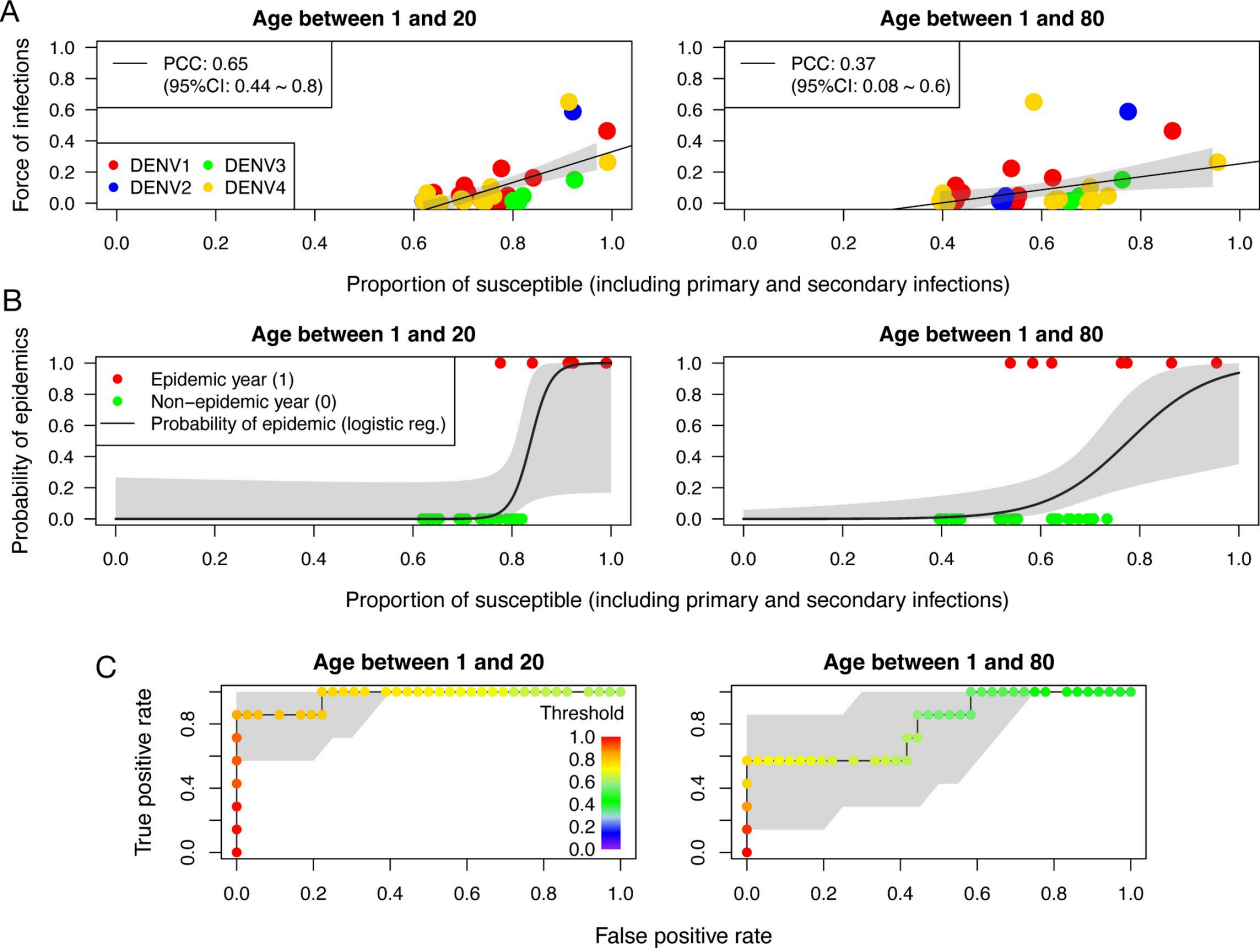
Relation between the proportion of susceptibles and FOI (obtained from our model). **(A)** FOI as a function of the fraction of the susceptibles to primary and secondary infections for children (left panel) and general population (right panel). Different colours represent a different circulating serotype. The black solid lines represent the linear regression to the data with 95%-CI as grey shaded areas. Pearson correlation coefficient (PCC) between the FOI and the fraction of the susceptible is also provided in the figure. **(B)** The probability of occurrence of an epidemic (black solid lines) as a function of the fraction of susceptibles. This probability is estimated using application of the logistic regression to the data (See Materials and methods Section). Grey shaded areas show 95%-CI. Red and green circles show epidemics and non-epidemic time periods, respectively. **(C)** ROC curve to illustrate the diagnostic ability of predicting an epidemic using the fraction of the population that are susceptible.

In Fig 5A we plot the FOI as a function of the fraction of susceptibles among children (left panel) and in the general population (right panel). Child susceptibility has stronger correlation with the FOI (Pearson Correlation Coefficient: 0.65; 95%-CI 0.44–0.80) than adult susceptibility (0.37; 95%-CI 0.08–0.60).

We next estimate the probability of occurrence of an epidemic as a function of the susceptible fraction by using logistic regression (See Section B in S1 Text for more details). Probabilities are plotted in Fig 5B for children (left panel) and for the general population (right panel). The probability sharply increases when the fraction of susceptibles among children exceeds 0.8. This suggests that this indicator might be used as an indicator for the occurrence of epidemics. To study the ability to predict an epidemic based on the fraction of susceptibles, we plot the Receiver Operating Characteristic (ROC) curve in Fig 5C, where True Positive Rates (TPR) are plotted as a function of False Positive Rates (FPR) for various threshold values for the fraction of susceptibles. We find that the closest point to the perfect classification (FPR = 0, TPR = 1) is the one using the threshold value 0.82–0.84, leading to FPR = 0 and TPR = 0.86. Similar graphs with different age stratifications (1–4, 5–9, 10–14, 15-) are shown in the Supporting Information (Fig B in S1 Text).

### Cross immunity and time-varying reporting probability

In the Supporting Information (Fig C in S1 Text), we compare the reporting probabilities inferred from our model with and without cross immunity. The reporting probability for DENV-1 secondary infection is 3.58 (95%-CI 2.20–5.38) times larger than for DENV-1 primary infection in the scenario with cross-immunity; but only 2.14 (95%-CI 1.03–3.47) times larger in the scenario without cross-immunity. This implies that without taking into account cross immunity, the reporting probability for secondary infections tends to be underestimated. Interestingly, we also find that the reporting probability for children aged 1–4 year old is estimated to be lower if cross immunity is not accounted for: The reporting probability is estimated at 0.75 (95%-CI 0.58–0.96) times that for those aged 5–9 y.o., compared to a value close to 1 when cross immunity is modelled.

Our model is robust against constraints we introduce concerning how the reporting probability changes in time as shown in the Supporting Information (Figs D, E, and F in S1 Text). There is a small tendency that the lower the variations in the reporting probability during the surveillance period, the larger the relative risk for secondary infections compared with primary infections, increasing slightly from 3.58 (95%-CI 2.20–5.38) to 3.96 (95%-CI 2.54–5.79) for DENV-1 if the model does not change at all the reporting probability over time.

### Evaluation of the inferential framework using synthetic data

**Fig 6:**
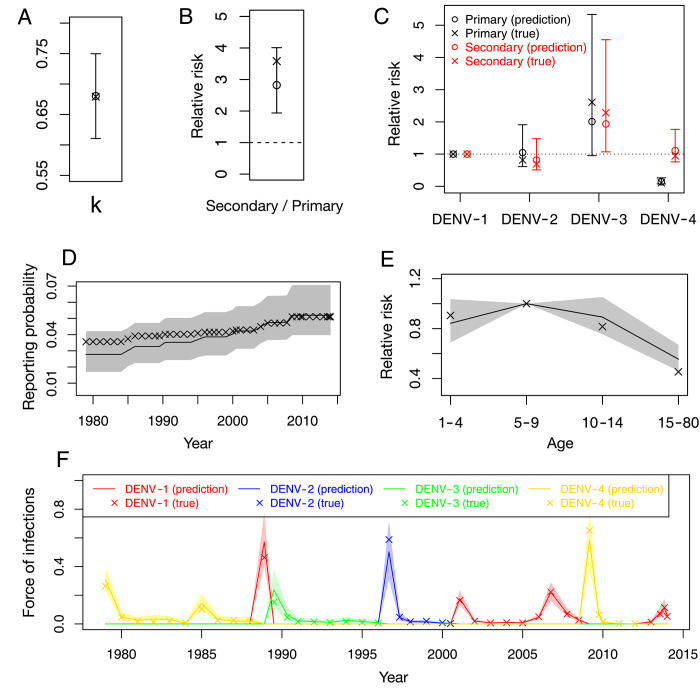
Evaluating the Bayesian inference using synthetic data. Using the model described in the main text, we generate synthetic data for given model parameters. Using this synthetic data, we then infer the model parameters using the Bayesian inference. The original parameters are plotted as crosses (true), while the inferred results are plotted as lines or circles with 95%-CIs (predictions). In (A), the parameter k for the negative binomial distribution is shown. In (B), the relative strength of the reporting probabilities of secondary infections (DENV-1) compared with primary infections (DENV-1) φ(1,2)/φ(1,1) is shown. In (C), the reporting probabilities relative to serotype 1, φ(i,1)/φ(1,1) for primary infections and φ(i,2)/φ(1,2) for secondary infections, are shown. Here i = 1,2,3,4 corresponds to DENV-i. In (D), the reporting probability of primary infections by DENV-1 T(t) is shown as a function of time. In (E), the age-factor of the reporting probability A(a) is shown. In (F), the FOI is shown as a function of time.

We evaluate our inferential framework. In Fig 6, we show the true parameters and the inferred results for the synthetic data with 95%-CIs. We observe that most of the true parameters are within the credible intervals (95%: 62 parameters out of 65), demonstrating the accuracy of our inferential framework.

## Discussion

In this article, we studied how the reporting probabilities of dengue infections depend on serotype, age, and the infection history of patients. To this goal, we generalised the sero-catalytic model with a reporting structure [10] by introducing reporting probabilities that vary with serotype, age, and the infection history of the patients. We fitted this model to case data in French Polynesia between 1979 and 2014 [11], where only mono-serotype circulations had been observed until the recent heterotypic outbreak 2013/2014, and to the result of the seroprevalence surveys conducted in 2014 and 2015 [13]. We estimated that the susceptible proportion dropped from 70% to 23% between 1979 and 2014, reflecting an increasing number of epidemics in recent years in French Polynesia.

The results also show that for DENV-1, the reporting probability for secondary infections is about three times higher than for primary infections. We estimated reporting probabilities for different serotypes and showed that DENV-3 infections were the most likely to be reported both for primary and secondary infections. In French Polynesia data, the DENV-3 epidemic and endemic transmission (1990–1996) happened just after the DENV-1 epidemic (1989). This sequence of events, DENV-1 (or DENV-2) followed by DENV-3, showed the highest severity in the Cuban epidemics [34, 35], consistent with our observations. The reporting probability for DENV-2 is similar to the one for DENV-1. This is in contrast to the observation that the secondary DENV-2 infection was 5–7 times more frequently associated with DHF than was secondary DENV-1 or DENV-3 infections in Cuba [35], although the sequence of events was DENV-3 followed by DENV-2 in our case, while it was DENV-1 followed by DENV-2 in the other case [35]. We note that reporting probabilities in our model do not necessarily reflect the probability of developing severe dengue infections, only that the infections are symptomatic enough to be detected by the surveillance system.

The epidemic of DENV-1 that took place in 2001 was followed by endemic circulation of DENV-1 for 5 years [11]. This then led to a new epidemic of DENV-1 in 2006. In the time-series analysis of the case data in different subdivisions [11], it was shown that DENV-1 circulated in the other subdivisions after the first epidemic, and the second epidemic in Windward islands was triggered because of the re-introduction of the virus from the Marquesas. Since our model only considers the Windward islands, we could not detect these inter-subdivision transmissions. Future studies could incorporate the spatial structure of the data and describe these inter-subdivison transmissions. Comparing changing patterns of reporting probabilities, as travel has become easier between the islands would be also interesting. We showed that the shape of the age distribution in Fig 4 was influenced by the year of previous epidemics. The epidemic in 2009 however needs further explanation because the plateau starts at 8 years old, even though, according to this argument, it should start at 3 years old as the previous epidemic by DENV-1 was in 2006. This indicates that those who drove the epidemic in 2006 were not small children less than 5 years old, but slightly older children, who lived through the 2001 epidemic.

In our model, we assumed that only a single serotype circulates at a given time. This assumption is valid before the 2013/2014 DENV outbreak [11]. To analyse data collected after this outbreak (and also hyperendemic regions other than French Polynesia), it would be necessary to take into account the concomitant circulation of different serotypes in our model. It would be also interesting to consider the seroprevalence of antibodies against Zika virus, which first emerged in French Polynesia in 2013 [13]. As there was no Zika circulation before this period, the data and our model could provide a fruitful test ground to study cross reactivity between Zika and DENV [36, 37].

Systematic deviations are observed for the seropositive fraction between the model estimation and the data. One possible reason for these deviations is from residual cross reactivity between DENV and Zika virus occurring during the final years of our surveillance period, which our model does not take into account. Another explanation could be the lack of case data before 1979. Our model infers the susceptible fraction by gradually reducing it from 1 starting from the year where the targeted population are born. Since the case data are not available before 1979, this indicates that the susceptible fraction of the adults older than 35 years old, who were born before 1979, is less accurate than those younger than 35 years old. Indeed, deviations in Fig 4B between the data and the model are greater for adults over 35 years old.

We assumed that the force of infection is constant over a year (or constant over each epidemic period), as our focus is on annual force of infection. It would be interesting in the future to consider a compartmental model where the force of infection can vary during an epidemic period.

The comparative study between our models with and without cross immunity suggested that the reporting probabilities for secondary infections tend to be underestimated without adding cross immunity to the model. On the other hand, we observed a small tendency that the lower the variations in the reporting probability during the surveillance period, the larger the relative risk for secondary infections compared with primary infections. These results imply a potential trade-off between modelling cross-immunity and modelling the variations of reporting probability over time.

Finally, the currently available licensed dengue vaccine, Dengvaxia, developed by Sanofi Pasteur, is recommended to be used on individuals who have already been infected by one of DENV serotypes [2]; otherwise, the vaccination can increase the risk of severe symptoms because of ADE [38]. By estimating the seroprevalence, our method could help to identify the region and also the age groups to be vaccinated in the presence of cross reactivities [10] as the WHO’s scientific advisory group of experts committee recommend the use of Dengvaxia only in places with the seroprevalence greater than 70% [39]. A new tetravalent dengue vaccine developed by Takeda (TAK-003) has been in phase 2 and 3 trials. The phase 2 trial involved the follow up of 1800 participants for 48 months and showed the absence of severe symptoms due to the vaccination of naive individuals [40]. There are still some concerns related to the efficacy of this vaccine against DENV 3 that might depend on the infection history of the individuals [41]. An additional 3-year period for long-term efficacy and safety evaluation is being conducted.

By analysing 35 years of dengue data in French Polynesia, we characterised key factors affecting the dissemination profile and reporting of dengue cases in an epidemiological context simplified by mono-serotypic circulation. Our analysis provides key estimates that can inform the study of dengue in more complex settings where the co-circulation of multiple serotypes can greatly complicate inference.

## Supporting information

S1 Text Supplementary Methods, Figures, and Tables. (PDF)
