# Supplementary material for: Reconstructing long-term dengue virus immunity in French Polynesia
Source: PLoS Negl Trop Dis. 2022 Oct 3;16(10):e0010367. doi: 10.1371/journal.pntd.0010367 (PMC9560594; doi:10.1371/journal.pntd.0010367)
Supplement: S1 Text — (PDF) [file pntd.0010367.s001.pdf]

Supporting Information for

Reconstructing long-term dengue virus immunity in French Polynesia

Takahiro Nemoto[1,2], Maite Aubry[3], Yoann Teissier[3,4], Richard Paul[5,6], Van-Mai Cao-Lormeau[3+*], Henrik Salje[1,7+], Simon Cauchemez[1+*]

1 Mathematical Modelling of Infectious Diseases Unit, Institut Pasteur, Université Paris Cité, UMR2000, CNRS, Paris, France.

2 Graduate School of Informatics, Kyoto University, Yoshida Hon-machi, Sakyo-ku, Kyoto, 606-8501, Japan

3 Laboratory of research on Infectious Vector-borne diseases, Institut Louis Malardé, French Polynesia.

4 Direction of Health, Ministry of Health, French Polynesia.

5 Functional Genetics of Infectious Diseases Unit, Institut Pasteur, Université de Paris, UMR2000, CNRS, Paris, France.

6 Pasteur Kyoto International Joint Research Unit for Integrative Vaccinomics, Japan.

7 Department of Genetics, University of Cambridge, Cambridge UK.

* mlormeau@ilm.pf, simon.cauchemez@pasteur.fr

+: Equal senior contribution

A. Derivations of Eqs. (1,2)

In this Supporting Information, we derive Eqs. (1,2) of the main text. We first focus on $x(t,a|\lambda)$. We consider the difference between $x(t+d,a+d|\lambda)$ and $x(t,a|\lambda)$ with a small increment d. Since this difference is the number of newly infected fraction of the population of age a at year t during a small increment d, it is written as

$$x(t+d,a+d|\lambda) - x(t,a|\lambda) = -d\, x(t,a|\lambda) \sum_{i=1}^{4} \lambda_i(t) \qquad (s1)$$

by definition of the force of infections. By dividing both sides by d, and taking the small d limit, we obtain

$$\frac{\partial}{\partial a} x(t,a|\lambda) + \frac{\partial}{\partial t} x(t,a|\lambda) = -x \sum_{i=1}^{4} \lambda_i. \qquad (s2)$$

It is straightforward to check if Eq. (1) in the main text is the solution of (s2).

Similarly, the difference between $y_i(t+d,a+d|\lambda)$ and $y_i(t,a|\lambda)$ is written as

$$y_i(t+d,a+d|\lambda) - y_i(t,a|\lambda) = d \left[x(t,a|\lambda)\lambda_i(t) - y_i(t,a|\lambda) \sum_{j\neq i} \lambda_i(t) \right], \qquad (s3)$$

where the first term in the right-hand side is the add to $y_i(t,a|\lambda)$ due to primary infections, while the second term is the removal from $y_i(t,a|\lambda)$ due to secondary infections. By dividing both sides by d, and taking the small d limit, we obtain

$$\frac{\partial}{\partial a} y_i(t,a|\lambda) + \frac{\partial}{\partial t} y_i(t,a|\lambda) = x(t,a|\lambda)\lambda_i(t) - y_i(t,a|\lambda) \sum_{j\neq i} \lambda_i(t). \qquad (s4)$$

Eq. (2) in the main text is the solution of this equation.

B. Visualisation of the inferred parameters

First of all, 95%-CIs used in this article are generated under the Bayesina framework using the negative binomial likelihood probability with the fitting parameter k that is inferred as 0.68 (95%-CI 0.61-0.75).

In Fig 2A, the relative strength of the reporting probabilities of secondary infections (DENV-1) compared with primary infections (DENV-1) is plotted. This is estimated as $\varphi(1,2)/\varphi(1,1)$. In Fig 2B, the reporting probabilities relative to serotype 1 are plotted for both primary and secondary infections. These are estimated as $\varphi(i,1)/\varphi(1,1)$ for primary infections and $\varphi(i,2)/\varphi(1,2)$ for secondary infections ($i = 1,2,3,4$). In Fig 2C, the time-dependent factor of the reporting probabilities $T(t)$ in Eq. (6) is plotted. In Fig 2D, the age factor of the reporting probabilities $A(a)$ in Eq. (6) is plotted.

In Fig 3B, the FOI $\lambda_i(t)$ is plotted ($i = 1,2,3,4$ corresponds to DENV-1, DENV-2, DENV-3, DENV-4). In Fig 3C, the average immunity profile of the population is plotted using the fitted parameters. The fraction of the never infected population averaged over age at the time t is estimated as $\int_1^{80} da\, x(t,a)p(t,a)$, where $p(t,a)$ is the normalised age distribution of the population size. The averaged fraction of the population who have

been infected once by a serotype i before the time t is estimated as $\int_1^{80} da\, y_i(t,a)p(t,a)$. Finally, the averaged fraction of the population who have been infected more than once before the time t is estimated as $\int_1^{80} da\, z(t,a)p(t,a)$.

In Fig 5A, FOI is plotted as a function of the fraction of the susceptible. Using the fitted model, this fraction (for circulating serotype i) is estimated as $\int_1^{A} da \left[x(t,a) + \sum_{j\neq i} y_j(t,a)\right] p(t,a)$ for each time period, where A is 20 for the children (the left panel of Fig 5A) and 80 for the general population (the right panel of Fig 5A). In Fig 5B, the probability of the occurrence of the epidemic (black solid lines) is plotted as a function of the fraction of the susceptible. This probability is estimated using logistic regression as detailed as follows: We first define the epidemic period as the period during which the reported number exceeds 300 (see Fig A). We then plot the value 1 as a function of the fraction of the susceptible for the epidemic periods (red filled circles in Fig 5B) and 0 for the non-epidemic periods (green filled circles in Fig 5B). Assuming a linear relationship between the fraction of the susceptible and the log-odds of the occurrence of epidemics, we determine this linear coefficient by fitting the corresponding Bernoulli sampling model to the 0-or-1 signal. For the ROC curve in Fig 5C, we first introduce a threshold value for the fraction of susceptible, above which we expect that the epidemic occurs and below which we do not. We then calculate the FPR (the rate at which an epidemic does not occur even if we expect it to occur) and the TPR (the rate at which an epidemic occurs as expected) for various values of the threshold. In Fig 5C, we plot filled circles at the position (x, y)=(FPR, TPR), where the colours of the filled circles correspond to the threshold value.

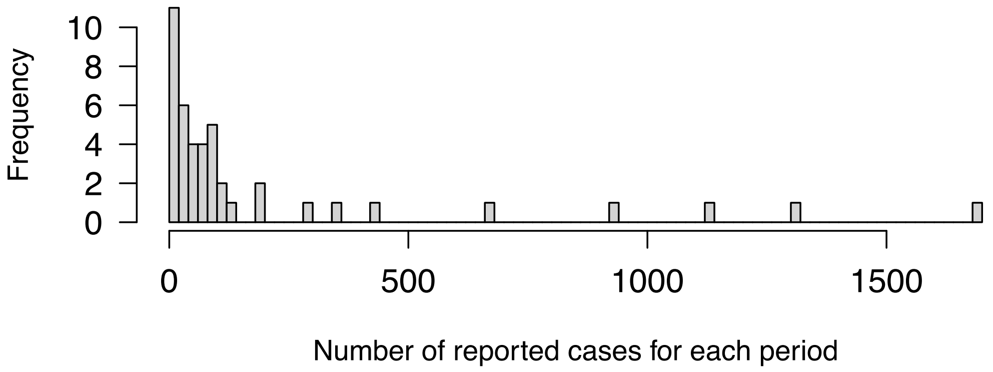

Fig A Histogram of the number of reported cases for each time period. *It shows a cluster of non-epidemic periods when the number of reported cases is below 250.*

Table A. Time periods where the force of infection takes a constant value.

Time period	Epidemic	Serotype
1 Jan 1979 - 31 Dec 1980	Yes	4
1 Jan 1980 - 31 Dec 1981	No	4
1 Jan 1981 - 31 Dec 1982	No	4
1 Jan 1982 - 31 Dec 1983	No	4
1 Jan 1983 - 31 Dec 1984	No	4
1 Jan 1984 - 31 Dec 1985	No	4
1 Jan 1985 - 31 Dec 1986	No	4
1 Jan 1986 - 31 Dec 1987	No	4
1 Jan 1987 - 31 Dec 1988	No	4
1 Jan 1988 - 24 Nov 1988	No	4
25 Nov 1988 - 2 July 1989	Yes	1
3 July 1989 - 30 April 1990	Yes	3
1 May 1990 - 31 Dec 1991	No	3
1 Jan 1991 - 31 Dec 1992	No	3
1 Jan 1992 - 31 Dec 1993	No	3
1 Jan 1993 - 31 Dec 1994	No	3
1 Jan 1994 - 31 Dec 1995	No	3
1 Jan 1995 - 31 Dec 1996	No	3
1 Jan 1996 - 1 Sep 1996	No	3
2 Sep 1996 - 30 April 1997	Yes	2
1 May 1997 - 31 Dec 1998	No	2
1 Jan 1998 - 31 Dec 1999	No	2
1 Jan 1999 - 31 Dec 2000	No	2
1 Jan 2000 - 1 July 2000	No	2
2 July 2000 - 29 Jan 2001	No	2

30 Jan 2001 - 31 Dec 2002	Yes	1
1 Jan 2002 - 31 Dec 2003	No	1
1 Jan 2003 - 31 Dec 2004	No	1
1 Jan 2004 - 31 Dec 2005	No	1
1 Jan 2005 - 31 Dec 2006	No	1
1 Jan 2006 - 1 Oct 2006	No	1
2 Oct 2006 - 1 Oct 2007	Yes	1
2 Oct 2007 - 1 July 2008	No	1
2 July 2008 - 27 Feb 2009	No	1
28 Feb 2009 - 1 Oct 2009	Yes	4
2 Oct 2009 - 31 Dec 2010	No	4
1 Jan 2010 - 31 Dec 2011	No	4
1 Jan 2011 - 31 Dec 2012	No	4
1 Jan 2012 - 31 Dec 2013	No	4
1 Jan 2013 - 31 July 2013	No	1
1 Aug 2013 - 30 Oct 2013	No	1
1 Nov 2013 - 31 Dec 2014	No	1
1 Jan 2014 - 23 Oct 2014	Yes	1

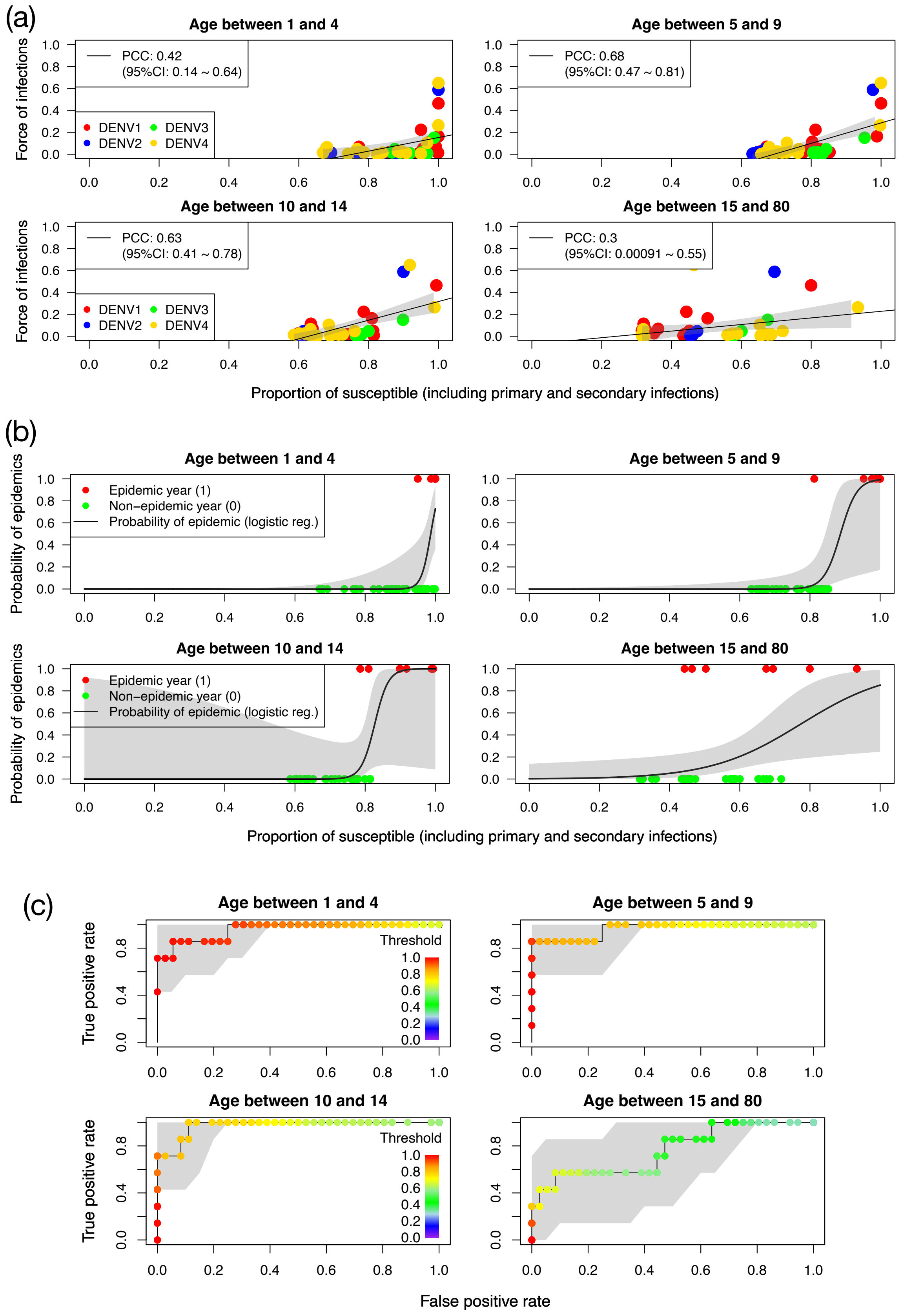

Fig B Relation between the proportion of susceptibles and FOI for age stratifications (1-4, 5-9, 10-14, 15-80). *In this figure, plotted quantities are the same as those in Fig 5 but with different age stratifications (1-4, 5-9, 10-14, 15-80).*

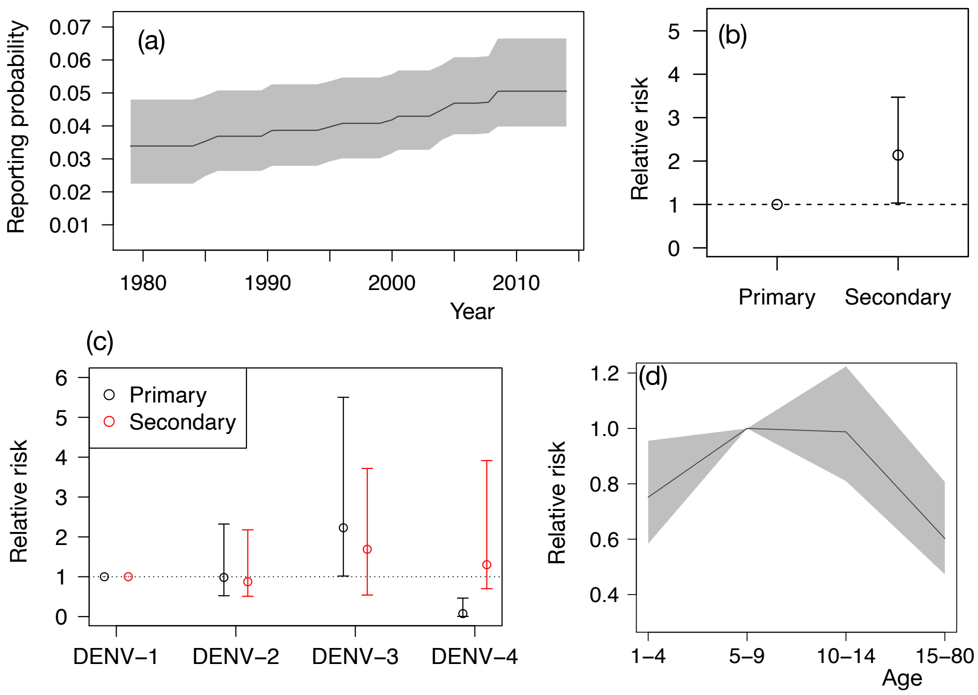

Fig C Estimated reporting probabilities for the model without cross immunity. *We consider the model without cross immunity, i.e.,* $I_s^{i,j}(a \mid \lambda)$ *is used in Eq.(7) instead of* $\overline{I_s^{i,j}}(a \mid \lambda)$, *and infer the reporting probabilities. Plotted quantities are the same as those in Fig 2.*

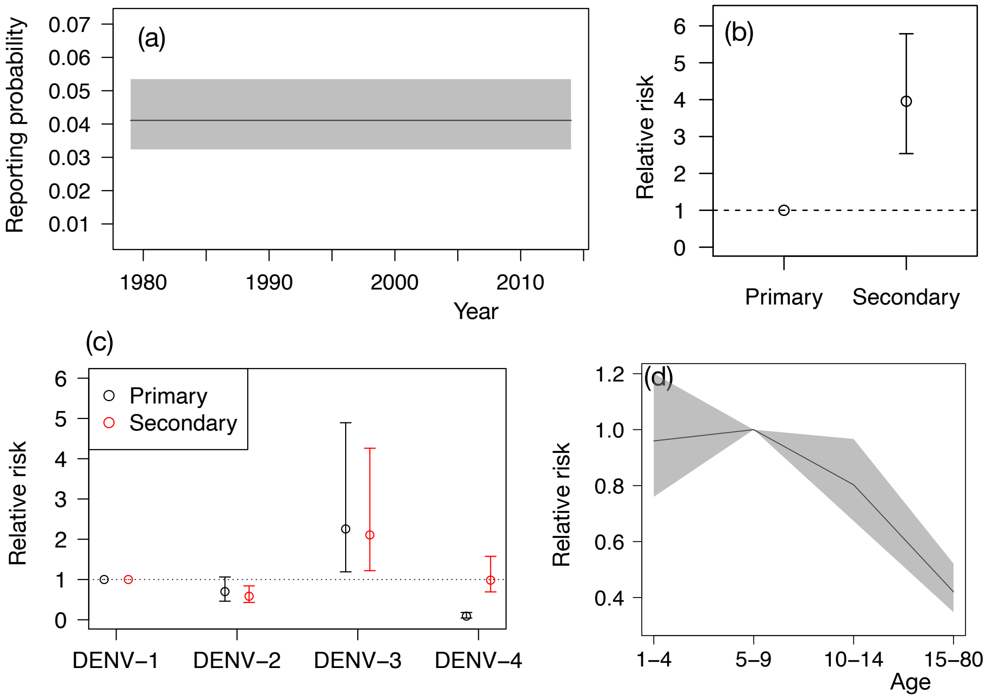

Fig D Estimated reporting probabilities for the model with constant reporting rates. *We consider the model with the constant reporting rate, i.e.,* $T(t)$ *in Eq.(6) is constant over time, and infer the reporting probabilities.*

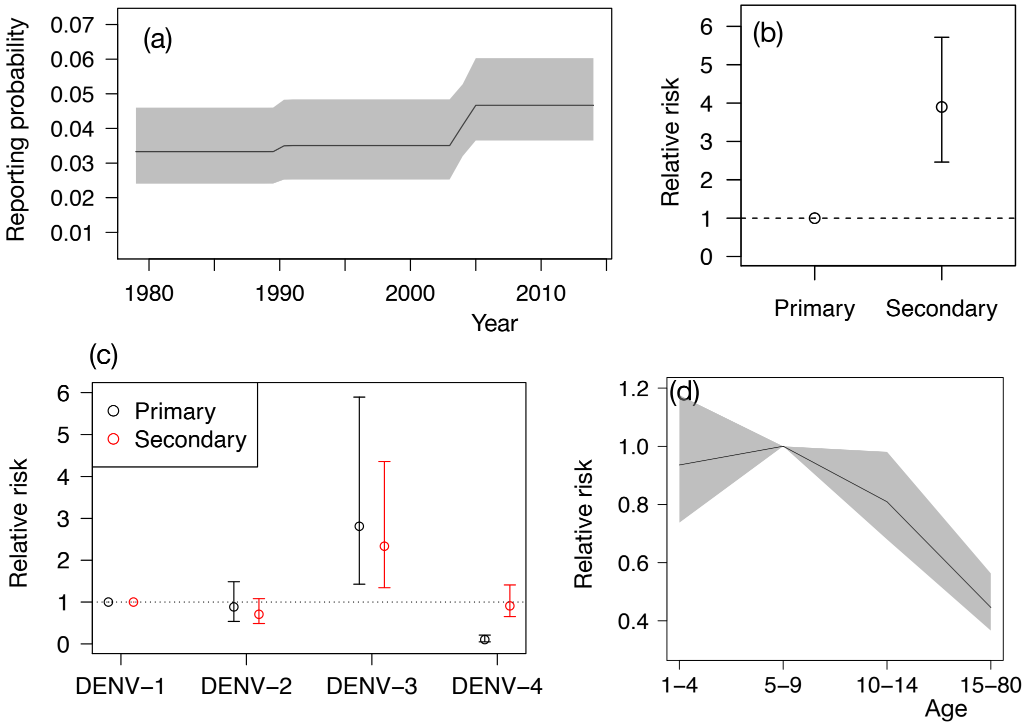

Fig E Estimated reporting probabilities for the model with less changing reporting probability over time (ver1). *We consider the model with $T(t)$ that is constant during 1979-1990, 1990-2004, 2005-2014, and infer the reporting probabilities.*

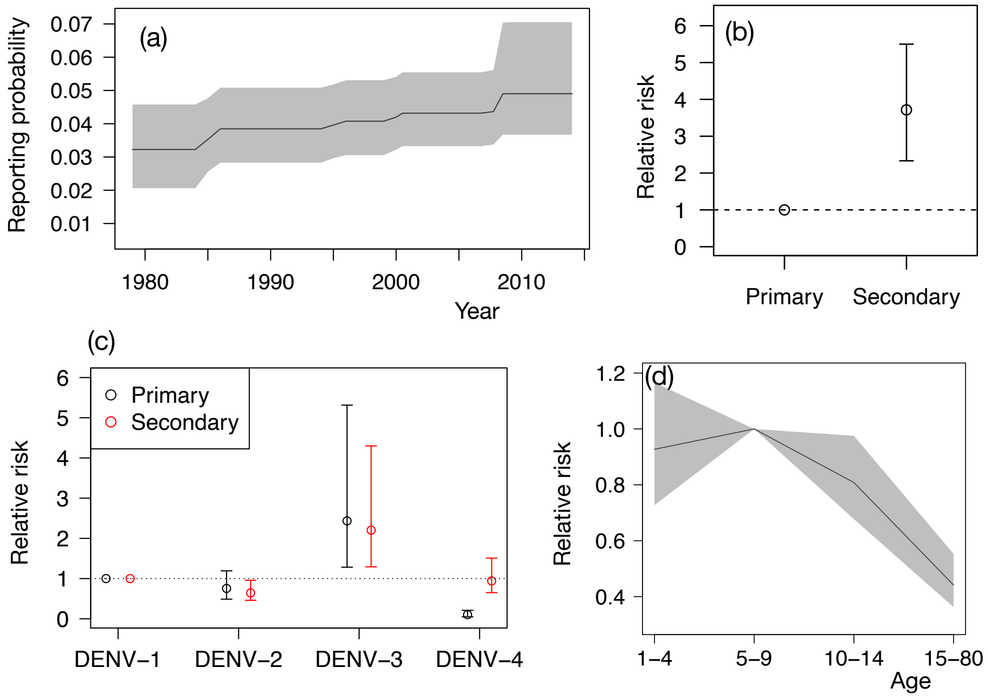

Fig F Estimated reporting probabilities for the model with less changing reporting probability over time (ver2). *We consider the model with $T(t)$ that is constant during 1979-1985, 1986-1995, 1996-2000, 2001-2008, 2009-2014, and infer the reporting probabilities.*
